# Disentangling the Benefits of Sex

**DOI:** 10.1371/journal.pbio.1001321

**Published:** 2012-05-01

**Authors:** Denis Roze

**Affiliations:** 1 CNRS, UMR 7144, Station Biologique de Roscoff, Roscoff, France; 2 UPMC University Paris 6, Roscoff, France

## Abstract

Understanding the evolutionary advantage of sexual reproduction remains one of the most fundamental questions in evolutionary biology. Most of the current hypotheses rely on the fact that sex increases genetic variation, thereby enhancing the efficiency of natural selection; an important body of theoretical work has defined the conditions under which sex can be favoured through this effect. Over the last decade, experimental evolution in model organisms has provided evidence that sex indeed allows faster rates of adaptation. A new study on facultatively sexual rotifers shows that increased rates of sex can be favoured during adaptation to new environmental conditions and explores the cause of this effect. The results provide support for the idea that the benefits of increasing genetic variation may compensate for the short-term costs of sexual reproduction.

## Abstract 2

In experiments with a facultatively sexual rotifer, populations adapting to novel environments evolve higher rates of sex because sexual mixing quickly assembles well-adapted genotypes.

## The Problem of Sex

Thinking about sex has been one of the main occupations of evolutionary biologists for almost half a century. The widespread occurrence of sexual reproduction—the recomposition of two parental genomes into the genome of a new individual—is indeed puzzling, given the important costs associated with this mode of transmission of genetic material (see Box 1). Despite that, complete asexuality is rare in the eukaryotic kingdom, and sex represents the only possible mode of reproduction in a substantial number of species (including ourselves). The oldest hypothesis on the evolutionary significance of sex was formulated by Weismann in 1889 [1] and elaborated during the first part of the 20th century by Morgan, Fisher, and Muller [2]–[4]: according to this hypothesis, sex is beneficial because it increases genetic variation, allowing faster rates of adaptation by combining different beneficial mutations into the same genome. However, following the seminal work of Maynard Smith and Williams in the early 1970s [5],[6], the apparent simplicity of this “Fisher-Muller” hypothesis was put into question: for sex to bring a net benefit, it must create advantageous genetic combinations more often than it destroys them. In population genetics terms, this implies the existence of “negative genetic associations” within populations: good alleles must tend to be associated with bad alleles at other loci, in which case sex can break these associations and generate genotypes combining beneficial alleles. But where do these negative associations come from? Different possible sources have been identified, corresponding to different theories to explain sex.

**Box 1. The Costs of Sex**

Many important costs are associated with sexual reproduction, in particular:

- **The cost of males (or “2-fold cost of sex”):** in many species, males do not provide any resource to the next generation, yet sexual females typically invest half of their resources into the production of males. Everything else being equal, this generates a 2-fold advantage for asexual females (producing only female offspring) [30].
- **The cost of breaking favourable genetic combinations:** genotypes that are able to survive to adulthood and reproduce prove that they are relatively fit in their own environment. Reproducing sexually may disrupt beneficial genetic combinations and lower the mean fitness of offspring.
- **Costs associated with the mating process:** finding a mate can be costly in time and energy and may also increase risks of predation and parasite transmission. Furthermore, in some species mating may harm the female and affect her future reproductive success.

First, certain forms of natural selection may generate such associations: this happens in particular when the advantage of beneficial alleles decreases as more beneficial alleles (at other loci) are added to the genome (or conversely when the effect of deleterious alleles increases as more deleterious alleles are added), a scenario described as *negative epistasis* [7]. Mathematical models show that in this situation, populations contain an excess of intermediate genotypes carrying a mixture of good and bad alleles. Increasing the rate of sexual reproduction increases the variance in fitness among offspring by creating extreme (both very good and very bad) genotypes. This is disadvantageous in the short term, because the high fitness of very good genotypes is not sufficient to compensate for the low fitness of very bad ones; however, it becomes advantageous in a longer term, because very good genotypes increase in frequency (carrying with them alleles that promote sex). When the direction of selection remains constant over time, the long-term benefit is stronger than the short-term cost only under restrictive conditions [8],[9] that do not correspond to observed patterns of epistasis [10]. The short-term cost may turn into a short-term advantage, however, when selection changes over time (as in some models of coevolution between species) or over space, generally making things easier for sex [11]–[16]. Finally, a different family of models has shown that chance events (that stem from the stochastic nature of mutation and individual reproduction in finite populations) also tend to produce negative genetic associations, thus generating an advantage for sex [17]–[22].

## Using Experimental Evolution to Explore the Benefits of Sex

For a long time, most of these theoretical models have been desperately crying for empirical validation. However, studies on real organisms (both in the lab and in natural populations) have been catching up, particularly during the last decade. In particular, several classical biological models proved very useful to explore the benefits of sex during adaptation, with different experimental evolution studies on *Chlamydomonas reinhardtii* [23], *Saccharomyces cerevisiae* [24], and *Escherichia coli* [25], demonstrating that sexual (or recombining) lines adapt faster to new environments than asexual lines. Can this translate into a net benefit for sexuals when competing against asexuals? Evidence for this has been recently provided by experimental populations of the nematode *Caenorhabitis elegans*, showing that this mostly self-fertilizing organism evolves towards higher rates of biparental sex when adapting to a new environment (or coevolving with a pathogen) [26],[27].

In this issue of *PLoS Biology*, a study by Becks and Agrawal [28] on monogonont rotifers goes one step further by dissecting the evolutionary advantage of sex during adaptation. Contrarily to their chaste cousins the bdelloids, which have been evolving without sex for several millions of years, monogonont rotifers are facultatively sexual, reproducing asexually at low density but switching to sexual reproduction in response to a chemical stimulus that indicates high density. In a previous study [29], the same authors showed that the propensity for sex of these rotifers (measured as their response to the sex-inducing stimulus) can evolve in laboratory populations and typically decreases under stable environmental conditions—indicating selection against sexual reproduction. This trend is much reduced, however, when populations are maintained in a heterogeneous environment (with restricted migration between two different habitats), suggesting that spatial heterogeneity in selection tends to favour sex.

For this new study, Becks and Agrawal use some of their previous populations that have been adapting to two environments (A and B) differing in their food composition and salinity. At the start of the experiment, 10 populations are transferred to the environment for which they are not adapted (5 from A to B, 5 from B to A), while 10 populations stay in their previous environment to serve as controls. The maladaptation of each of the transferred populations to its new environment is clearly shown by an initial crash in density and a drop in fitness, measured by individual fitness assays; however, after 50 days both density and fitness have returned to their original level (before transfer), providing clear evidence for adaptation. What about sex? As in their previous study, the authors observe a steady decline of the propensity for sex within control populations. In both types of adapting populations, however, the propensity for sex increases, before reaching a plateau and declining as the population becomes adapted. Several lines of evidence indicate that this response is genetic rather than plastic—in particular, no change is observed if the opportunity for selection for sex is removed by forcing individuals into clonal reproduction. Selection for sex is further demonstrated by comparing the fitness of individuals derived from sexually versus asexually produced eggs (sampled directly from the populations): sexually derived individuals have a much lower fitness than asexually derived ones in control populations, but this pattern reverses during adaptation.

**Figure 1:**
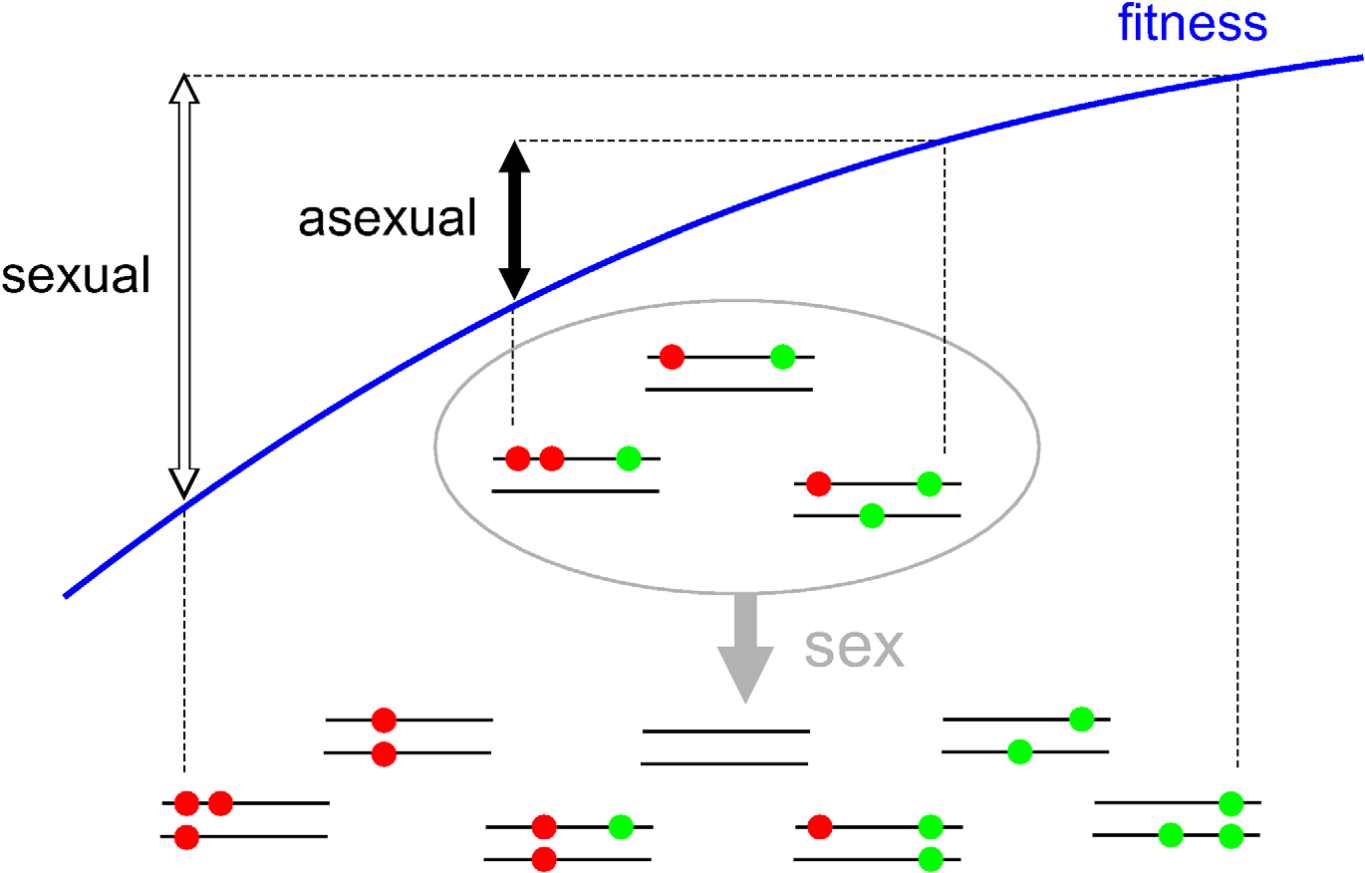
A genetic interpretation of the effect of sex on the fitness of offspring. Different diploid genotypes are represented by pairs of horizontal bars, where red and green dots represent deleterious and beneficial mutations, respectively. The initial population consists of the genotypes within the grey circle; asexual reproduction regenerates the same genotypes, while sex generates new genotypes by recombination and segregation. Because the initial population harbours negative genetic associations (excess of genotypes combining good and bad alleles, either at the same or at different loci), sex increases the variance in fitness among offspring. Although fitness is lower on average among sexually produced individuals than among asexually produced ones (due to the negative curvature of the fitness function), the best genotypes are sexually produced. These genotypes will increase in frequency, carrying along alleles that increase the rate of sex.

In order to obtain a deeper understanding of the mechanism generating selection for sex, Becks and Agrawal perform a last experiment: they collect random samples of rotifers from the different treatments at different time steps and force them into either sexual or asexual reproduction (by exposing them to a very strong sex-inducing stimulus or keeping them at low density). The results are particularly illuminating: in all treatments, sexually derived individuals display a lower mean fitness but a higher variance in fitness than asexually derived ones. The lower mean fitness demonstrates a short-term cost for sexual reproduction, both in control and adapting populations. The increased variance reflects the fact that sex tends to break negative genetic associations, resulting in a higher proportion of high- and low-fitness genotypes (see Figure 1): in particular, in the case of adapting populations the top-end of the fitness distribution reaches higher values among sexually-produced offspring, driving the evolution of increased rates of sex in these populations. This is the first experimental demonstration of the basic tenet of Weismann's hypothesis (that sex can be favoured because it increases genetic variation).

## Prospects

This study opens the door to a series of questions concerning the genetic mechanisms underlying these effects of sex on the mean and variance in fitness. First, what are the relative effects of inter-locus and intra-locus genetic associations? As illustrated by Figure 1, the increased fitness variance due to sex may stem from the fact that recombination between genomes carrying beneficial and deleterious alleles at different loci results in the production of genomes combining different beneficial alleles, or different deleterious alleles. Under this scenario, the fact that sex decreases the mean fitness of offspring would be indicative of epistatic interactions among loci, generating a negative curvature of the fitness function. Alternatively, Figure 1 also shows that the same effects may arise if sex tends to create homozygous individuals from heterozygous parents (allowing in particular the production of individuals carrying beneficial alleles in the homozygous state); here, the short-term cost of sex could be explained by dominance effects between alleles at the same locus—for example, due to the unmasking of recessive deleterious alleles. Both scenarios share the same prerequisite, however, which is the initial presence of negative genetic associations: excess of genotypes combining beneficial and deleterious alleles, either at the same or at different loci. This brings up a second question: what generates these negative associations? As stated earlier, two possible sources have been identified by theoretical studies: the deterministic action of selection and stochastic effects due to finite population size. For example, if heterozygotes at a given locus have a higher fitness than the average of both homozygotes, selection tends to produce an excess of these heterozygotes, which is maintained across generations when reproduction is partly clonal [9]. Alternatively, an excess of heterozygotes may stem from the fact that in any finite population, new mutations first appear in the heterozygous state (and are maintained heterozygous as long as reproduction is clonal [18]). Answering these questions will require other carefully planned experiments, comparing evolutionary responses at different population sizes and exploring the genetic architecture of fitness variation within experimental populations.

To what extent can the benefit of sex demonstrated here compensate for the strong costs associated with sexual reproduction in natural populations? Interestingly, some of these direct costs operate in Becks and Agrawal's experiment (the cost of males in particular). Nevertheless, one may object that the observed increase in the propensity for sex stays modest and is only transient. Is adaptive change sufficiently frequent under natural conditions to maintain a strong positive pressure on sexual reproduction? Comparisons of fitness distributions of sexually versus asexually produced individuals in natural populations would represent an important indication but remain scarce. Furthermore, even some of the most basic aspects of natural selection remain poorly known: does selection typically remain constant over long time periods, or does it fluctuate rapidly? What maintains variation for fitness between individuals? Does adaptive change typically involve a large number of genes, or only a few? Answering these different questions still represents a formidable task, but will be undoubtedly facilitated by recent technologies. Because it is so intimately linked with these fundamental issues, solving the problem of sex will ultimately require a deeper empirical knowledge of the evolutionary process in general. In the meantime, experimental evolution will remain an invaluable tool for assessing the plausibility of theoretical scenarios.
